# Knowledge, perceptions, and behavior regarding children’s oral health among Syrian pediatricians: a cross-sectional study

**DOI:** 10.1186/s12903-023-03022-x

**Published:** 2023-05-10

**Authors:** Mawia Karkoutly, Ammar Kataish, Saleh Al Kurdi, MHD Bashier Almonakel, Nada Bshara

**Affiliations:** 1 Pediatric Dentistry Department, Faculty of Dentistry, Dental College, Damascus University, Damascus, Syrian Arab Republic; 2 Department of Pediatrics, Faculty of Medicine, Damascus University, Damascus, Syria

**Keywords:** Pediatricians, Knowledge, Perceptions, Practices, Children, Oral health, Questionnaire

## Abstract

**Background:**

Oral health has a direct impact on health-related quality of life (HRQoL). Hence, general health and oral health cannot be separated. Pediatricians play a pivotal role in providing primary care for infants and are the first to interact with them since birth. Therefore, the aim of this study was to evaluate the knowledge, perceptions, and behavior of Syrian pediatricians regarding children’s oral health.

**Methods:**

This was a cross-sectional study. The questionnaire consisted of six main sections and required answers regarding demographic characteristics, knowledge, behavior, perceptions, and training received in oral health. The association between categorical variables was tested with Pearson’s chi-square tests and Fishers exact tests using SPSS ver. 23.

**Results:**

The response rate was 43.12% (229/531). Most of the participants (64.02%) got a poor level of knowledge and no significant association was found between knowledge level and years of experience (*p* = 0.270). The majority of the participants (99.13%) perceived that diet, bacteria, and sugar exposure time are the etiological factors of dental caries. The overwhelming majority of the participants (98.25%) acknowledged the need for further oral health training during residency.

**Conclusions:**

Most pediatricians reported a poor level of knowledge. It is recommended to update the postgraduate and residency curriculum to equip pediatricians with adequate knowledge regarding children’s oral health.

## Background

Oral health is a fundamental component of a child’s well-being and growth [1]. Evidence has linked poor oral health and unmet dental needs to other serious systemic conditions such as respiratory diseases, stroke, diabetes, cardiovascular diseases, and iron deficiency [2]. In addition, oral health has a direct impact on health-related quality of life (HRQoL) [3]. Therefore, general health and oral health cannot be separated [4].

Early childhood caries (ECC), is the presence of one or more decayed, missing, or filled primary tooth surfaces in preschool children. ECC is a major worldwide oral health problem with high prevalence in middle eastern countries [5]. Before the Syrian crisis, the prevalence and severity of ECC had been growing, it was estimated to be around 81% in 2011. During the crisis, no formal epidemiological studies have been conducted [6]. However, the protracted displacement of the Syrian refugee could lead to oral health deterioration [7]. In addition, dental caries was highly prevalent among Syrian children aged 8–12 years old residing in Damascus [8]. ECC results in poor sleep patterns, learning disabilities, poor quality of life, and growth disorders in toddlers due to the associated pain [5]. According to Jackson et al. [9], poor oral status has a negative impact on school performance and results in school absence.

Pediatricians play a pivotal role in providing primary care for infants and are the first to interact with them since birth. Parents often have several visits to the pediatrician in order to provide medical care, monitor child development, and administer vaccines [10]. Therefore, pediatricians should have vast knowledge regarding caries risk assessment, oral health screening, and caries prevention strategies. Unfortunately, pediatricians receive inadequate training regarding oral health during their residency according to several international surveys [11]. Therefore, the aim of this study was to evaluate the knowledge, perceptions, and behavior of Syrian pediatricians regarding oral health. Such studies identify knowledge gaps and highlight the necessity of updating the curriculum of pediatric residents and providing training for pediatricians regarding oral health.

## Material and methods

This was a cross-sectional survey research design. Ethical approval was provided by the Research and Ethics Committee of Damascus University (N 219/2022). Participation was voluntary and anonymous. An online Arabic questionnaire was designed using Google Forms software and adapted from previously validated questionnaires [12–14]. To determine its reliability, a Cronbach’s alpha test was performed after distributing it to a group of 20 participants in an interval of 2 weeks and comparing their answers for each question. It has shown an acceptable up to good values. The sample size was determined according to Krejcie and Morgan table in accordance with the total number of pediatricians (531). It was first piloted by a group of 10 pediatricians to ensure comprehensibility and clarity, and no additional changes were needed. It was then distributed to the pediatricians’ members of Damascus Medical Association (*n* = 531) via email and social networks. The questionnaire was online for three months; between September and December 2022.

The questionnaire consisted of six main sections. The first section obtained the sociodemographic data of the participants regarding gender, age, years in practice, and work sector. The second section covered participants’ knowledge regarding primary dentition. The third section consisted of nine questions addressing different participants’ behavior regarding dental practices and preventive measures for toddlers. The fourth section covered participants’ perceptions concerning dental caries and etiological factors. The fifth section consisted of three questions related to participants’ attitude toward receiving training during their residency. The last section included four illustrations related to clinical cases regarding dental caries and enamel defects used in a similar questionnaire [12].

The participants’ level of knowledge was scored based on the American Academy of Pediatric Dentistry (AAPD) recommendations [15]. The scoring was “0” for each incorrect answer and “1” for each correct answer, this was for questions related to the second and the last section. The level of knowledge was divided into three categories:

- Knowledge score < 50% = poor knowledge level
- 50% ≤ Knowledge score ≤ 75% = fair knowledge level
- Knowledge score > 75% = satisfactory knowledge level

Google Forms data was exported to an Excel spreadsheet (Microsoft Excel, Microsoft Corp, WA, USA) and then statistical analysis was done using IBM SPSS software v. 23 (IBM Corp., Armonk, USA). Descriptive statistics were calculated (frequency and percentage). The association between categorical variables (knowledge level and years in practice) was determined by using Pearson’s chi-square tests and fishers exact tests. The statistical significance was adjusted at 0.05 (*p* < 0.05).

## Results

Out of 531 pediatricians invited to participate, only 232 responded. Three questionnaires were excluded due to their missing answers and the response rate was 43.12% (229/531).

### Demographic characteristics of the participants

Almost two third (66.81%) of the participants were female. More than three quarters (83.41%) of the pediatricians were aged 21–29 years, with less than 10 years of experience (79.47%) and worked in the public sector (48.90%) (Table 1).

**Table 1:** Demographic characteristics of the participants

Characteristic	*N* (%)
Gender	
Male	76 (33.19)
Female	153 (66.81)
Age group	
21–29 years	191 (83.41)
30–39 years	12 (5.24)
40–49 years	10 (4.37)
50–59 years	13 (5.67)
> 60 years	3 (1.31)
Years in practice	
< 10 years	182 (79.47)
11–20 years	16 (6.98)
21–30 years	19 (8.29)
31–40 years	10 (4.36)
> 40 years	2 (0.87)
Work sector	
Public	112 (48.90)
Private	51 (22.27)
Combined	66 (28.82)

### Participants’ knowledge regarding oral health

**Fig. 1:**
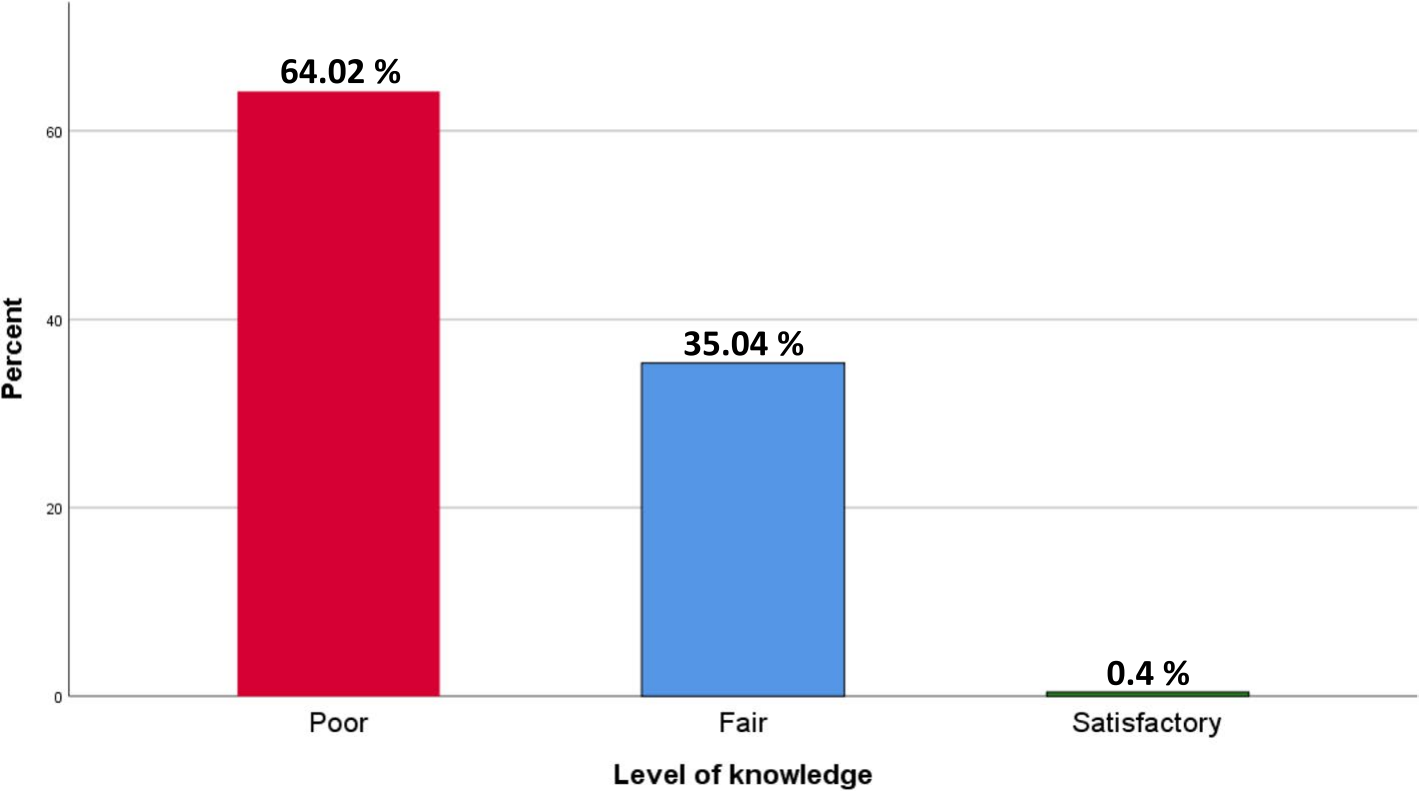
Participants’ level of knowledge (%) regarding oral health

The majority of pediatricians knew the right time for the first primary tooth eruption (85.15%) and the total number of primary teeth as well (60.26%). Approximately, half of the participants (46.72%) indicated that the optimal time to use fluoride toothpaste is when the child is effectively able to brush their teeth. About a third of pediatricians (34.93%) recommended the first dental visit by 1 year of age. The majority of the participants (79.48%) believed that dental caries is a non-transmissible disease. A minority of participants (15.72%) knew that prolonged breastfeeding is an etiological factor in tooth decay. More than half of the pediatricians (57.64%) considered that parents should supervise brushing until the child can do it alone. More than three-quarters of the participants (81.66%) do not know the correct concentration of fluoride toothpaste recommended for children under 6 years of age, while only a few (4.80%) knew (Table 2). Regarding lesion illustrations, participants could correctly identify only 2 cases (Table 3). Most of the participants (64.02%) got a poor level of knowledge (Fig. 1), and no significant association was found between knowledge level and years of experience (*p* = 0.270) (Table 4).

**Table 2:** Knowledge regarding oral health

Questions	*N* (%)
When does the first primary tooth erupt?	
4–5 months	29 (12.66)
**6–8 months**	195 (85.15)
12 months	4 (1.75)
15 months	1 (0.44)
Number of primary teeth:	
25	4 (1.75)
28	64 (27.95)
**20**	138 (60.26)
18	23 (10.04)
When should you brush your child’s teeth with fluoride toothpaste?	
**As soon as the first tooth erupts**	42 (18.34)
When several teeth have erupted	41 (17.90)
When the child is able to use the brush	107 (46.72)
I am not sure	39 (17.03)
First visit to the dentist:	
6 months of age	29 (12.66)
**1 years old**	80 (34.93)
When the first cavities are present	40 (17.47)
3 years old	80 (34.93)
Is dental caries a transmissible disease?	
Yes	34 (14.85)
**No**	182 (79.48)
I do not know	13 (5.68)
Do you believe that breastfeeding is an etiological factor in tooth decay?	
**Yes**	36 (15.72)
No	164 (71.62)
I do not know	29 (12.66)
Up to what age should parents supervise brushing?	
Until the child can do it alone	132 (57.64)
Up to 3 years of age	11 (4.80)
**Up to 7–8 years of age**	86 (37.55)
Never	0 (0.00)
For children under 6 years of age, what concentration of fluoride toothpaste do you recommend?	
1450 ppm F	2 (0.87)
**1000 ppm F**	11 (4.80)
500 ppm F	29 (12.66)
I do not know	187 (81.66)

Correct answers are written in bold

**Table 3:**
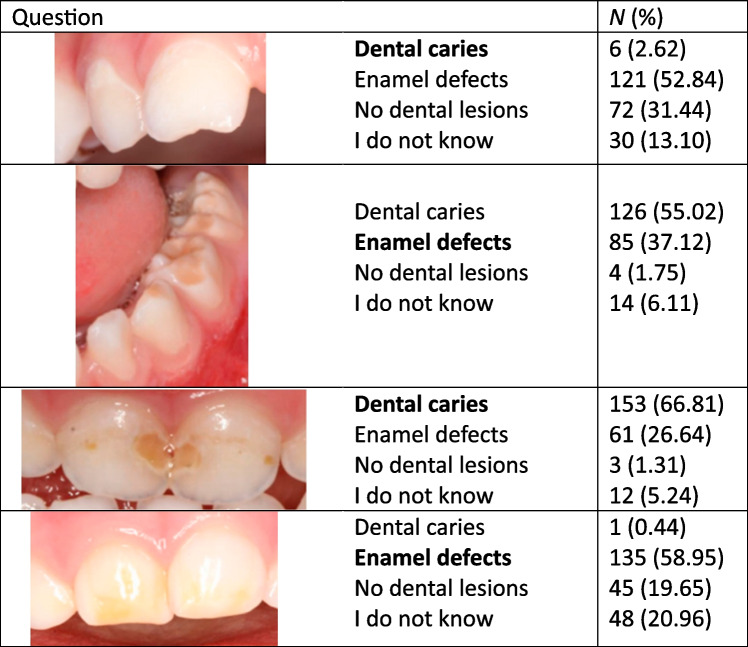
Lesion identification by clinical imaging

Correct answers are written in bold

### Participants’ perceptions regarding dental caries and etiological factors

The majority of the participants (99.13%) perceived that diet, bacteria, and sugar exposure time are the etiological factors of dental caries. Furthermore, most of them perceived that avoiding non-natural juices (90.39%), and sugary drinks (88.65%) are the main restrictions on sugary food (Table 5).

**Table 4:** Association between the level of knowledge and pediatricians’ years in practice

Years in practice	Poor level of knowledge *N* (%)	Fair level of knowledge *N* (%)	Satisfactory level of knowledge *N* (%)	*p*-value
< 10 years	120 (65.93)	61 (33.52)	1 (0.55)	0.409
11–20 years	11 (68.75)	5 (31.25)	0 (0.00)	
21–30 years	7 (36.84)	12 (63.15)	0 (0.00)	
31–40 years	7 (90.00)	3 (10.00)	0 (0.00)	
> 40 years	2 (100.00)	0 (0.00)	0 (0.00)	

**Table 5:** Perceptions regarding dental caries and etiological factors

Questions	*N* (%)	
Etiological factors: (multiple choice)	Yes	No
Diet and bacteria	227 (99.13)	2 (0.87)
Hereditary factor	194 (84.72)	35 (15.28)
Fluoride	127 (55.46)	102 (44.54)
Sugar exposure time	227 (99.13)	2 (0.87)
Saliva	125 (54.59)	104 (45.41)
Restrictions on sugary foods: (multiple choice)		
No snacking	112 (48.91)	117 (51.09)
Do not drink non-natural juices	207 (90.39)	22 (9.61)
Avoid sugary drinks	203 (88.65)	26 (11.35)
Avoid sweets	24 (10.48)	205 (89.52)

### Participants’ behavior regarding oral health

Almost half of the participants (46.72%) always examined the patient’s teeth from the first year of life. More than half of the participants (51.53%) occasionally provided oral health education to parents. The majority of the participants (86.03%) referred patients to a pediatric dentist only when parents indicate dental problems. Around two-thirds of participants (66.81%) occasionally remarked on the cariogenic effects of going to bed with a bottle. More than half of the participants (58.08%) instructed their patients on how to brush their teeth. Regarding oral hygiene recommendations, almost a third of the participants (34.50%) believed that the use of dental floss is age dependent. Furthermore, 37.99% of the participants occasionally recommended the use of fluoride toothpaste while about half of them (53.28%) never recommended the use of fluoride mouth rinses under 6 years of age. In addition, the majority of them (73.80%) never prescribed fluoride tablets or drops for children under 3 years of age (Table 6).

**Table 6:** Behavior regarding oral health

Questions	*N* (%)
Do you examine the patient’s teeth from the first year of life?	
Always and routinely	107 (46.72)
Only if pain is reported	42 (18.34)
Occasionally	76 (33.19)
Never	4 (1.75)
Do you provide oral health education to parents?	
Always and routinely	101 (44.10)
Occasionally	118 (51.53)
Never	10 (4.37)
When do you refer your patients to a pediatric dentist?	
Always and routinely	27 (11.79)
When parents indicate dental problems	197 (86.03)
Never	5 (2.18)
Do you remark on the cariogenic effects of going to bed with a bottle?	
Always and routinely	63 (27.51)
Occasionally	153 (66.81)
Never	13 (5.68)
Do you indicate how to brush your patients’ teeth?	
Always and routinely	65 (28.38)
Occasionally	133 (58.08)
Never	31 (13.54)
Do you recommend the use of dental floss?	
Always and routinely	27 (11.79)
Occasionally	48 (20.96)
Never	75 (32.75)
Depend on age	79 (34.50)
Do you recommend fluoride toothpaste?	
Always and routinely	61 (26.64)
Occasionally	87 (37.99)
Never	16 (6.99)
Depend on age	65 (28.38)
For children under 6 years of age, do you recommend the use of fluoride mouth rinses?	
Always and routinely	2 (0.87)
Occasionally	53 (23.14)
Never	122 (53.28)
Depend on age	52 (22.71)
Do you prescribe fluoride tablets or drops for children under 3 years of age?	
Always	2 (0.87)
Occasionally	58 (25.33)
Never	169 (73.80)

### Participants’ training received in oral health

The majority of the participants (92.83%) indicated that they had not received any pediatric dentistry training during their residency and 95.96% of them believed that the level of training devoted to oral health is inadequate. The overwhelming majority of the participants (98.25%) acknowledged the need for further oral health training during residency (Table 7).

**Table 7:** Participants’ training received in oral health

Questions	*N* (%)
Have you received any pediatric dentistry training during your residency?	
Yes	17 (7.17)
No	207 (92.83)
Was the level of training devoted to oral health adequate?	
Yes	9 (4.04)
No	214 (95.96)
Do you acknowledge the need for further oral health training during residency?	
Yes	225 (98.25)
No	4 (1.74)

## Discussion

Although oral health is a fundamental component of a child’s growth [1], to the best of our knowledge, this is the first questionnaire to investigate knowledge, perceptions, and practices regarding children’s oral health among Syrian pediatricians. An online questionnaire was used due to its accessibility to the targeted population [16].

Most of the pediatricians (85.15%) are able to identify the optimal time for the first primary tooth eruption, and the total number of primary teeth as well (60.26%). This result is in accordance with the finding reported in Spain [12]. AAPD recommends the first dental visit by the first birthday [15]. In this present questionnaire, about a third of pediatricians (34.93%) believed that the ideal time for the first dental visit is at 1 year of age. However, their peers in the United Arab Emirates (UAE) reported better knowledge and a higher percentage of 51.4% [17]. World Dental Federation (FDI) has declared that dental caries is a noncommunicable disease (NCD) [18]. Most of the participants (79.48%) knew that dental caries is a non-transmissible disease.

According to National Health Service (NHS) guidelines, parents should supervise brushing until at least the age of seven [19]. A possible explanation for this is that elementary school children (≥ 6 years old) have adequate motor skills to brush their teeth independently [20]. In the present study, more than half of the participants (57.64%) believed that parents should supervise brushing until the child can do it independently regardless of their chronological age. The overwhelming majority of the pediatricians (81.66%) were unaware of the correct concentration of fluoride toothpaste recommended for children under 6 years of age. This finding is not surprising as most pediatricians got a poor level of knowledge regarding oral health. This could be due to the inadequate training devoted to oral health during pediatricians’ residency. However, their peers in neighboring Lebanon and Kuwait reported an acceptable level of knowledge [21, 22].

Regarding lesion identification, the vast majority of the pediatricians (97.38%) could not identify initial caries in the deciduous incisor. The early detection of white spot lesions or “nonexistence cavitations” is difficult yet important before they progress into irreversible cavities [23]. A similar result was reported in Spain, the UK, Brazil, and Trinidad and Tobago [12, 24–26]. However, more than half of them (66.81%) were able to identify the irreversible cavities. Concerning enamel defect lesions diagnosis, pediatricians were able to identify them in molars rather than incisors. This finding is in agreement with the one reported in Spain [12]. However, enamel defect diagnosis is challenging even for dentists [27].

Most of the participants (86.03%) referred patients to a pediatric dentist only for treatment not for applying preventive measures. However, according to AAPD recommendations, oral screening, dietary consultation, and preventive measures should be provided since the eruption of the first primary tooth [15]. Approximately two-thirds of the pediatricians (66.81%) occasionally remarked on the cariogenic effects of nighttime bottle feeding. This could be clarified by the well-known fact that falling asleep with a milk bottle leads to ECC [28]. Furthermore, a minority of participants (15.72%) knew that prolonged breastfeeding could cause dental caries. According to Peres et al. [29], prolonged breastfeeding for more than two years leads to severe early childhood caries (S-ECC). Around half of the participants (53.28%) never recommended the use of fluoride mouth rinses under 6 years of age. According to Marinho et al. [30], fluoride mouth rinses are not recommended for children under the age of six due to the risk of accidental swallowing, and dental fluorosis occurrence. Approximately three-quarters of the pediatricians (73.80%) never prescribed fluoride tablets or drops under the age of three. However, according to Tubert-Jeannin et al. [31], little is known about the efficacy of fluoride tablets or drops in tooth caries prevention for children under the age of six. In addition, there is no sufficient evidence to prove fluoride supplements’ safety. Those two previous findings are similar to those reported in Spain [12].

The overwhelming majority of the participants (99.13%) perceived that diet, bacteria, and sugar exposure time are the etiological factors of tooth decay. Moreover, most of them believed that avoiding non-natural juices (90.39%), and sugary drinks (88.65%) are the main restrictions on sugary food. Unfortunately, according to Almonakel et al. [32], the most consumed snacks among Damascus children are biscuits, chips, and chocolate, which are rich in carbohydrates. Prolonged exposure to carbohydrates leads to dental caries due to acidic plaque formation [33].

Almost all participants (98.25%) acknowledged the need for further oral health training during residency. This reflects the urgent need to update the curriculum of medical schools to equip pediatricians with further knowledge regarding oral health.

Interprofessional collaboration between pediatricians and pediatric dentists is crucial for achieving optimal health. Pediatricians can play a critical role in identifying early signs of dental caries. They can collaborate with pediatric dentists to provide education on caries factors, and preventive measures. In addition, children with special health care needs and certain medical conditions may require additional care from both a pediatrician and a pediatric dentist. Overall, pediatricians should refer patients to pediatric dentists if they believe that specialized dental care is necessary for the child’s overall health and well-being [34]. Hence, it is recommended to update the postgraduate and residency curriculum to equip pediatricians with adequate knowledge regarding oral and dental practices.

Despite the good response rate (43.12%), this study has limitations. First, the use of a close-ended and self-administered questionnaire may not reflect the actual beliefs of the participants [35]. Second, most of the pediatricians had fewer than 10 years of experience.

## Conclusions

Most pediatricians reported a poor level of knowledge and requested further training regarding oral health. No association was found between the level of knowledge, perceptions of Syrian pediatricians and their skill to prevent or maintain children oral health in good state.

## Data Availability

The datasets generated during and/or analysed during the current study are available from the corresponding author on reasonable request.

## Abbreviations

HRQoL: Health-related quality of life
ECC: Early childhood caries
AAPD: American academy of pediatric dentistry
UAE: United Arab Emirates
FDI: World dental federation
NCD: Noncommunicable disease
NHS: National health services
UK: United Kingdom
S-ECC: Severe early childhood caries
